# Gene selection for studying frugivore-plant interactions: a review and an example using Queensland fruit fly in tomato

**DOI:** 10.7717/peerj.11762

**Published:** 2021-08-05

**Authors:** Shirin Roohigohar, Anthony R. Clarke, Peter J. Prentis

**Affiliations:** School of Biology and Environmental Science, Queensland University of Technology (QUT), Brisbane, Queensland, Australia

**Keywords:** Quantitative reverse transcription PCR, Gene expression, Fruit induced-defense, Frugivorous insect, Detoxification genes, Tephritidae

## Abstract

Fruit production is negatively affected by a wide range of frugivorous insects, among them tephritid fruit flies are one of the most important. As a replacement for pesticide-based controls, enhancing natural fruit resistance through biotechnology approaches is a poorly researched but promising alternative. The use of quantitative reverse transcription PCR (RT-qPCR) is an approach to studying gene expression which has been widely used in studying plant resistance to pathogens and non-frugivorous insect herbivores, and offers a starting point for fruit fly studies. In this paper, we develop a gene selection pipe-line for known induced-defense genes in tomato fruit, *Solanum lycopersicum,* and putative detoxification genes in Queensland fruit fly, *Bactrocera tryoni,* as a basis for future RT-qPCR research. The pipeline started with a literature review on plant/herbivore and plant/pathogen molecular interactions. With respect to the fly, this was then followed by the identification of gene families known to be associated with insect resistance to toxins, and then individual genes through reference to annotated *B. tryoni* transcriptomes and gene identity matching with related species. In contrast for tomato, a much better studied species, individual defense genes could be identified directly through literature research. For *B. tryoni*, gene selection was then further refined through gene expression studies. Ultimately 28 putative detoxification genes from cytochrome P450 (P450), carboxylesterase (CarE), glutathione S-transferases (GST), and ATP binding cassette transporters (ABC) gene families were identified for *B. tryoni*, and 15 induced defense genes from receptor-like kinase (RLK), D-mannose/L-galactose, mitogen-activated protein kinase (MAPK), lipoxygenase (LOX), gamma-aminobutyric acid (GABA) pathways and polyphenol oxidase (PPO), proteinase inhibitors (PI) and resistance (R) gene families were identified from tomato fruit. The developed gene selection process for *B. tryoni* can be applied to other herbivorous and frugivorous insect pests so long as the minimum necessary genomic information, an annotated transcriptome, is available.

## Introduction

Tephritid fruit flies are globally significant pests of horticulture (Aluja & Mangan, 2008; Qin et al., 2015). Frugivorous tephritids lay their eggs into fruit, where the resultant larvae hatch and feed, causing yield loss (Hafsi et al., 2016). With a global trend in trying to reduce the use of pesticides for insect pest control (Ricroch, 2019), alternative management strategies for fruit flies are needed (Sarwar, 2015). Plant breeding for fruit fly resistance is one such option, and while this might be achieved through traditional selection methods (Choudhary et al., 2018; Medjkouh et al., 2018), it is more likely to be achieved in the modern era through manipulation of the plant’s defense genes using biotechnological tools (Kumar et al., 2020).

At the phenotype level, numerous studies have documented how variation between fruit, at the species, variety and ripening-stage levels, can impact on fruit fly offspring survival (Aluja, Díaz-Fleischer & Arredondo, 2004; Nunes et al., 2015; Roohigohar, Prentis & Clarke, 2020). Some of these studies have also correlated fruit fly offspring performance with fruit traits such as peel toughness (Díaz-Fleischer & Aluja, 2003; Rattanapun, Amornsak & Clarke, 2009), amount of peel oils and secondary chemicals (Papachristos, Papadopoulos & Nanos, 2008; Papachristos et al., 2009), or forming calluses in ‘Hass’ and ‘Sharwil’ avocados around *Anastrepha* sp. egg clusters using a combination of chemically and mechanically induced resistance mechanisms in fruit (Aluja et al., 2014). However, the amount of genotypic data available to help understand fruit fly/fruit interactions to progress biotechnology-based plant defense breeding is sparse and limited to a molecular study of induced defenses of green olive drupes against the olive fruit fly, *Bactrocera oleae* (Corrado et al., 2012; Grasso et al., 2017). This lack of genetic information is in contrast to insect folivory research (Dugé de Bernonville et al., 2017; Gloss, Abbot & Whiteman, 2019; Subramanyam et al., 2019), and also plant pathogen research where there is a growing body of molecular data on fruit defense (Alkan et al., 2015; Rao & Nandineni, 2017; Baba et al., 2019).

Different analytical techniques can be applied to the study of molecular interactions between fruit and frugivorous insects. Corrado et al. (2012) and Grasso et al. (2017) applied comparative transcriptomic and proteomics in their studies, which provides a comprehensive overview of the molecular and protein responses associated with the interaction. However, a limitation of this approach, particularly in non-model organisms (such as crop pest species), is the inability to ascribe function to non-annotated genes and proteins (Giron et al., 2018; Kumaran et al., 2018). A complementary approach can be achieved through gene expression analyses to examine the expression of specific genes already known to be associated with plant/herbivore interactions (Zheng & Dicke, 2008). One of the most reliable techniques for gene expression studies is quantitative reverse transcription PCR (RT-qPCR) (Prasch & Sonnewald, 2013).

The RT-qPCR approach has been used to provide insight to plant defense pathways and insect detoxification gene expression during plant/herbivore interactions in several systems (De Oliveira, Pallini & Janssen, 2019; Altuntaş, Duman & Kılıç, 2020; Dixit et al., 2020; Quais et al., 2020). For example, in fruit/pathogen interaction studies, the over-expression of 10 phenylpropanoid genes in orange fruit infested by *Penicillium digitatum* which led to changes in the metabolite profile of the fruit (Ballester, Lafuente & González-Candelas, 2013) was determined using RT-qPCR; while in apples infested with *Penicillium expansum* RT-qPCR was used to track upregulation of defense-related genes and reactive oxygen species genes (Vilanova et al., 2014). Similarly, RT-qPCR has been used to document the over-expression of stress perception genes such as *Prosystemin* in tomato and tobacco plants in response to *Manduca sexta* larval feeding (Orozco-Cardenas, McGurl & Ryan, 1993; Gilardoni et al., 2011); and the upregulation of direct defense genes such as *CYP79B2/B3* in *Arabidopsis* and *TD* gene in tomato plant tissue against *Spodoptera exigua* larval feeding (Müller et al., 2010; Gonzales-Vigil et al., 2011). In insects, RT-qPCR has also been used to demonstrate the upregulation of known detoxification genes *Slgstel, Cyp321a7, Cyp321a9* and *Cyp6ab14* which increased *Spodoptera litura* larval resistance against plant toxic allelochemicals (Wang et al., 2015a; Wang et al., 2015b; Zou et al., 2016; Wang et al., 2017a; Wang et al., 2017b).

**Figure 1:**
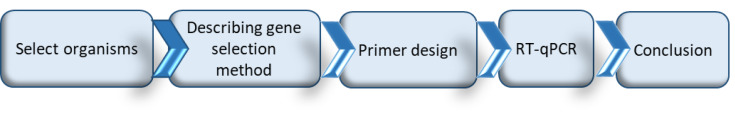
Schematic representation of the process followed to undertake a RT-qPCR study, and the structural outline of the following sections of this paper.

While a valuable counterpart to untargeted transcriptomic and proteomic studies, carrying out a RT-qPCR study from the beginning is not trivial. Before initiating such a study, it first needs to be determined if enough is already known about the system to support such an approach, if so then what are the appropriate genes for study, PCR primers need to be developed for those genes, the appropriate experiments and subsequent RT-qPCR analyses have to be undertaken, and then the results analyzed (Fig. 1). In this paper we work through the RT-qPCR developmental pipeline for a specific fruit fly/fruit system (Queensland fruit fly in tomato fruit) focusing particularly on the selection of appropriate herbivore-induced fruit defense genes and insect detoxification and sequestration genes. While doing so we identify generic issues for consideration to facilitate other RT-qPCR studies in fruit flies and their host fruit, and present a review on the metabolic pathways and associated genes known to be linked with fruit defense and insect detoxification.

## Materials and Methods

### Choosing organisms for study

#### The frugivore: Queensland fruit fly-*Bactrocera tryoni*

Selection of the study organism should be driven by research priority, but ideally also the ability to extrapolate results across to related organisms and the availability of some existing genomic information. In Australia, *Bactrocera tryoni* (Froggatt) is a highly polyphagous horticultural pest, attacking most fleshy vegetables and fruit crops (Clarke et al., 2011), and so there is a strong local need for research on this organism. While locally important, *B. tryoni* can also serve as a suitable model species for other tephritids as the biology of related species is quite similar (Clarke, 2019). Published transcriptomes of *B. tryoni* are available (Gilchrist et al., 2014; Kumaran et al., 2018) and there is a close similarity of the genetics on this fly and congeneric species. For example, according to the National Centre for Biotechnology Information (NCBI) database, putative *B. tryoni* detoxification pathway genes have an above 90% identity match with *Bactrocera dorsalis* (Hendel), one of the world’s most destructive agricultural pests (Qin et al., 2018).

#### The fruit: tomato-*Solanum lycopersicum*

We chose tomato, *Solanum lycopersicum*, as our model fruit for a number of reasons which should be considered when thinking about what fruit type to use. Firstly, tomato has an accessible, fully sequenced genome (Tomato-Genome-Consortium, 2012) with numerous related genetic and genomic resources available through the National Center for Biotechnology Information (NCBI) database. Its genome is also relatively small (950 Mb) and, conveniently for genomic research, is a diploid species (Gerszberg et al., 2015). Tomato can be grown under many different cultivation conditions (from fully-controlled environments to open-field) with a relatively short life-cycle and has well documented and accessible cultivar variation. These attributes make it an already well-established model system for the study of plant/pathogen and plant/herbivore molecular interactions (Rodriguez-Saona et al., 2010; Kawazu et al., 2012). Finally, for our work, it was already known that different tomato cultivars and ripening stages have significant phenotypic effects on *B. tryoni* offspring performance (Balagawi et al., 2005; Roohigohar, Prentis & Clarke, 2020), and we hypothesized that the difference in performance has a molecular basis.

### Strategy in choosing genes of interest

**Figure 2:**
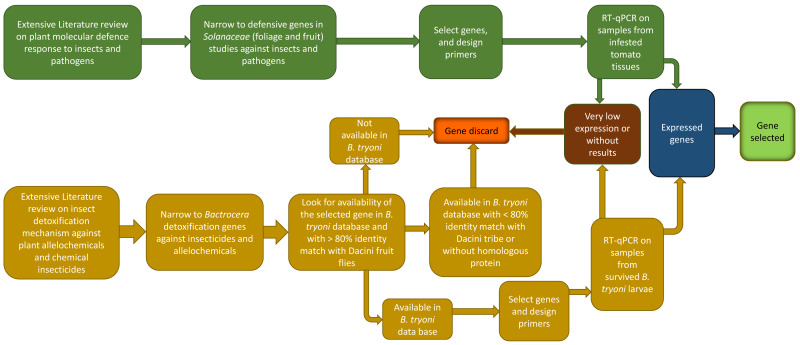
Schematic representation of the workflow used in the present study to choose inducible defense-related genes in tomato fruit and detoxification-related genes in* Bactrocera tryoni*.

The process for selecting genes of interest is summarized in Fig. 2. Gene selection is a sequential process that involves a combination of literature research, PCR primer design, and laboratory testing. Not all candidate genes identified through literature research may end up being selected because of bioinformatic limitations, or because the genes themselves have very low expression in preliminary trials. The process to identify *B. tryoni* detoxification genes and tomato defense genes follow.

#### *Bactrocera tryoni* detoxification genes

Larvae of tephritids such as *B. tryoni*, must stay in a single fruit to complete development (Fitt, 1984); thus, they may utilise specific molecular mechanisms to detoxify fruit toxic secondary compounds. Understanding which molecular pathways fruit fly larvae use to survive in fruit is an essential component when studying frugivore-fruit molecular interactions. In the absence of prior studies on *B. tryoni* larval detoxification genes, we selected target genes using four steps (Fig. 2): (i) a comprehensive literature review on insect detoxification mechanisms against plant allelochemicals and chemical pesticides; (ii) an exhaustive review of any similar studies in other tephritids; (iii) searching for detoxification genes in the *B. tryoni* functional annotation database (Kumaran et al., 2018); and (iv) checking nucleotide and peptide sequences in NCBI-BLAST database, tblastn, blastx, blastp, smartblast, and Universal Protein Resource/Uniprot to check for homologous proteins, protein domains and genes with >80% identity match from species within the tribe Dacini, such as *Bactrocera dorsalis*. Insects mostly utilize the same enzymes for detoxification of plant allelochemicals and insecticides (Dai et al., 2019), hence searching for genetic information from both plant allelochemical and insecticide studies is appropriate.

**Figure 3:**
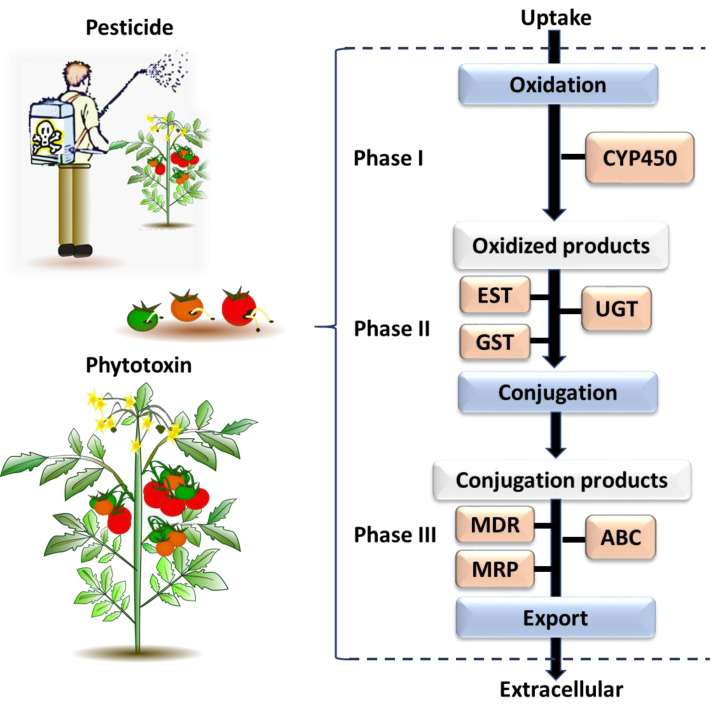
The general process of chemical detoxification by insects. Phase I and Phase II involve metabolizing enzymes altering toxic chemicals, while Phase III involves transport and export of conjugated metabolites and elimination of toxins pre-biotransformation. The major gene families associated with each phase are followed: CYP450, Cytochrome P450; EST, Carboxylesterase; GST, Glutathione S-transferase; UGT, UDP-Glucosyltransferase; MDR, Multidrug Resistance Protein; MRP, Multidrug Resistance Associated Protein; ABC, ATP Binding Cassette (Berenbaum & Johnson, 2015; Saha, 2016).

The genes identified through this process were associated with different phases of the insect chemical-detoxification process, which occurs for both the detoxification of plant secondary chemicals and pesticides (Heidel-Fischer & Vogel, 2015; Heckel, 2018). During Phase I, genes/enzymes such as P450 monooxygenases (P450s) and carboxylesterases (COEs) are involved in oxidation, hydrolysis or reduction of toxic compounds; subsequently, Phase II involves the conjugation of the modified toxins with hydrophilic groups such as glutathiones, sulphate and sugars by glutathione S-transferases (GSTs) and UDP-glucosyltransferase (UGT) to enhance the polarity of the molecules and so help excretion; while finally, in Phase III, ATP-binding cassette transporters export the conjugated toxins out of the cell (Donkor et al., 2019). Each phase of the detoxification process is associated with major gene families (Fig. 3). The following section describes each of selected gene families, and then provides a list of the individual selected genes for *B. tryoni* larvae. Not all potential genes identified through initial literature searching progressed to the selection stage. Listing all discarded genes is space prohibitive, but for illustrative purposes a selection of the excluded genes, and why they were discarded, are shown as a Data S1.

#### PHASE I

##### Cytochrome P450 (P450s).

Cytochrome P450 monooxygenases (cytochrome P450s) are a large, complex, and highly conserved gene family of heme-thiolate proteins that encode P450 enzymes (Feyereisen, 2006). The P450s contribute to the catalysis of numerous oxidative reactions during endogenous and exogenous metabolism (Li & Liu, 2019). Numerous studies report the important role of P450 genes in the metabolism of xenobiotics (George, Rao & Rahangadale, 2019) and plant allelochemicals (Hazzouri et al., 2020).

In Lepidoptera, the members of the P450 CYP6 subfamily play a crucial role in detoxifying a variety of plant toxic secondary compounds (Li, Berenbaum & Schuler, 2001). In Hemiptera, over-expression of the *CYP6CY3* gene in *Myzus persica* helped to detoxify nicotine from the tobacco plant (Bass et al., 2013). In *B. dorsalis*, high expression of *CYPA41* and *CYP6EK1* in the larval and adult malpighian tubules suggest their potential role in detoxification of pesticides (Huang et al., 2012). Amongst 12 P450 genes, *CYP6D9*, *CYP12C2*, and *CYP314A1* were upregulated in *B. dorsalis* following insect exposure to malathion and beta-cypermethrin; while *CYP4E9* expression was upregulated in response to abamectin and beta-cypermethrin exposure (Huang et al., 2013). Insecticide resistance linked to higher expression of P450 genes and their related enzymes has been proposed for both *B. dorsalis* (Jing et al., 2020) and *B. oleae* (Pavlidi et al., 2013).

For *B. tryoni* larvae, 20 genes were selected from the cytochrome P450 gene family: *CP6A9, CP313, CP134, CP4D8, CP6G1, C12E1, CP6T1A, CP6T1B, C12C1, C12B1, C12B2, CP304A, C304B, CP306, C6A14, C4AC2, CP4S3, CP132, CP316* and *CP6G2.*

#### PHASE II

##### Carboxylesterase (CarEs).

Carboxylesterase (CarEs) are a multigene superfamily ubiquitous in almost all organisms (Marshall et al., 2003). CarEs are involved in hydrolyzing a broad range of ester-containing xenobiotics such as drugs, environmental toxicants, and insecticides (Feng, Li & Liu, 2018). Studies on insect CarEs are mainly focused on their role in metabolizing insecticides and differential expression of CarE genes has been associated with insecticide resistance in different number of insects (Farnsworth et al., 2010; Wang et al., 2019).

CarE genes are associated with the development of malathion resistance in *B. dorsalis* (Wang et al., 2015a; Wang et al., 2015b; Wang, 2016), with the functional role of the esterase B1 (*BdB1*) gene strongly confirmed (Wang et al., 2017a; Wang et al., 2017b). In *B. oleae*, 15 CarE genes were identified as being involved in the metabolism of plant phytotoxins and insecticides (Pavlidi et al., 2013). Two genes from the CarEs superfamily in *B. tryoni* larvae were selected: *ESTF* and *EST1.*

##### Glutathione S-transferase (GST).

Glutathione S-transferases (GSTs) are another multigene family, present in most organisms, which are associated with detoxification (Hayes, Flanagan & Jowsey, 2005). In insects, GSTs have a diversity of functions such as participation in olfaction, oxidative stress responses, and the development and bioactivation of ecdysteroids and hormones (Enya et al., 2015; Zhao et al., 2020), but they are mainly associated with detoxification of endogenous and xenobiotic compounds (Enayati, Ranson & Hemingway, 2005; Che-Mendoza, Penilla & Rodríguez, 2009). The upregulation of GSTs and insecticide resistance is well documented (Shi et al., 2012; Zhang et al., 2019), as is the association between GSTs and insect detoxification of plant allelochemicals (Mittapalli, Neal & Shukle, 2007; Huang et al., 2011).

In *B. oleae*, 33 GSTs are involved with the metabolism of xenobiotics, such as chemical insecticides and plant phytotoxins (Pavlidi et al., 2013). GSTs activities were significantly higher in malathion and *λ*-cyhalothrin treated *B. zonata* (Yaqoob et al., 2013); while overexpression of the GST gene *BdGSTd9* has been identified as a component of malathion resistance in *B. dorsalis* (Meng et al., 2020).

Three genes from the GST superfamily identified for *B. tryoni* larvae were *GSTD1, GSTT1* and *GSTT7*.

#### PHASE III

##### ATP binding cassette (ABC) transporters.

ATP binding cassette (ABC) transporters are one of the largest transporter gene families across the metazoans (Xiao et al., 2018). ABCs are found in almost all organisms, where they typically have a role in the ATP-dependent transport of various substrates across biological membranes (Broehan et al., 2013). Most ABC transporter genes encode membrane-bound proteins which transport a wide range of molecules, such as amino acids, peptides, sugars, vitamins, sterols, lipids, hormones, endogenous metabolites, inorganics and xenobiotics, across membranes (Dean, Hamon & Chimini, 2001). Studies of the physiological functions of ABC transporters in arthropods are limited to only a few species (Xiao et al., 2018), typically the “model” species such as *D. melanogaster*, *B. mori*, *Anopheles gambiae*, *Apis mellifera*, and *Tribolium castaneum* (Roth et al., 2003; Liu et al., 2011; Broehan et al., 2013), but large numbers of genes are known to be involved in ABC transporter pathway (Rösner & Merzendorfer, 2020).

The role of ABC transporters in efflux pumps, facilitating cellular excretion of insecticides or metabolites, strengthens a hypothesis for their playing a role in insecticide resistance in insects (Rösner & Merzendorfer, 2020). In a recent study of *S. litura* resistance to pyrethroid, the *ABC5* gene was significantly upregulated and showed a strong correlation with insecticide resistance (Xu et al., 2020). In *B. dorslis*, ABC transporter genes might play roles in the insecticide resistance, with several *bdABC* genes significantly upregulated after treatment of *B. dorsalis* with malathion, abamectin, and beta-cypermethrin (Xiao et al., 2018). In *B. oleae*, 18 ABC transporter genes were reported for their possible roles in handling xenobiotics, such as plant phytotoxins and insecticides (Pavlidi et al., 2013).

Five genes from the ABC transporters family were selected from *B. tryoni* larvae: *ABCG1, ABCA3, SUR, L259* and *MDR49*.

#### Selection of tomato defense genes

**Figure 4:**
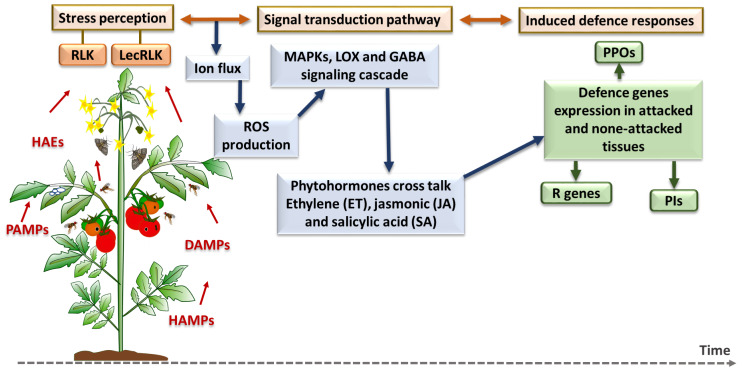
Plant inducible defense responses against arthropod and pathogen stressors. At the stress perception stage, DAMPs = damage-associated molecular patterns; HAEs = herbivore-associated elicitors; HAMPs = herbivore-associated molecular patterns; and PAMPs = pathogen-associated molecular patterns. The major defense pathways and defense gene families are as follow: RLK, Receptor-Like Kinase; LecRLK, Lectin Receptor-Like Kinase; ROS, Reactive Oxygen Species; MAPK, Mitogen-Activated Protein Kinase; LOX, Lipoxygenase; GABA, Gamma-Aminobutyric Acid; PPO, Polyphenol Oxidase; R genes, Resistance genes; PI, Protease Inhibitor (Santamaria et al., 2013; Santamaria et al., 2018).

Genes associated with induced-defense responses in tomato were mostly selected based on previous studies of *Solanaceae*–insect/pathogen molecular interactions; either in plant vegetative tissue or fruit. While defense genes in tomato are much better known than putative detoxification genes in *B. tryoni*, a literature review on tomato/herbivore and tomato/pathogen molecular interactions was still needed to ensure an appropriate selection of genes from across different defense pathways (Fig. 4).

Plant induced defense responses towards herbivores and pathogens first requires recognition systems, such as receptor-like kinase (RLK) and lectin receptor-like kinase (LecRLK), that can perceive herbivore-associated elicitors (HAEs), herbivore-associated molecular patterns (HAMPs), damage-associated molecular patterns (DAMPs) and pathogen-associated molecular patterns (PAMPs) (Santamaria et al., 2013). This recognition triggers the plant’s cell defense responses which are started by ion fluctuation across the membrane and production of reactive oxygen species (ROS), and then continued by mitogen-activated protein kinase (MAPK) cascades phosphorylation and responses, lipoxygenase (LOX) pathway or GABA signalling pathway stimulation (Nejat & Mantri, 2017). After specific phytohormonal crosstalk among a plant’s essential defense-related phytohormones, which include salicylic acid (SA), jasmonic acid (JA) and ethylene (ET) as the core components of plant immune system (Tsuda & Katagiri, 2010), plant defense genes and enzymes such as polyphenol oxidase (PPOs), and protease inhibitors (PIs) are expressed and accumulate in damaged and undamaged plant tissue (Liu, 2018). In the next section, a brief description of each defense pathway and the related gene families are provided before the introduction of individual genes.

#### Plant perception

##### Receptor-Like kinase.

A plant’s perception systems allow them to detect physical injury and pest chemical elicitors through the use of specific receptors, such as receptor-like kinases (RLKs) (Santamaria et al., 2018). RLKs are composed of a transmembrane region, an intracellular kinase domain, and an ectodomain that potentially contributes to ligand binding (Macho & Zipfel, 2014). Plant perception of phytophagous arthropod attack through RLKs has been predominantly investigated in lepidopteran and aphid attack on vegetative tissue (Gouhier-Darimont et al., 2013; Gouhier-Darimont et al., 2019), and pathogen attack to both vegetative (Hashemi et al., 2020) and fruit tissues (Haile et al., 2019).

The target genes associated with RLKs in tomato were PEPR1/2 Ortholog Receptor-Like Kinase1 (*PORK1*) and Lectin receptor kinase1 (*LecRK1*)*.*

The *PORK1* gene (also known as the Tomato Protein Kinase1b (*TPK1b*) interacting protein, (Xu et al., 2018)) has biological functions in wound-systemin signalling and systemin-mediated plant responses to both fungal infestation and insect attack (Liu et al., 2013; Klauser et al., 2015), where systemin is a polypeptide hormone unique to, but common within the Solanaceae (Ryan & Pearce, 2003). *PORK1* is a key determinant of systemin responses in tomato with an important role in tomato plant resistance to *B. cinerea* fungi and *M. sexta* larvae (Xu et al., 2018). *LecRK1* gene activity is important in suppressing insect-mediated inhibition of jasmonic acid-induced defense responses in *Nicotiana attenuata* during herbivory by *M. sexta* larvae (Gilardoni et al., 2011); while conversely suppressing the expression of *LecRK1* in *N. attenuata* increased *M. sexta* folivory (Bonaventure, 2012).

#### Plant defense signalling transduction

##### D-mannose/L-galactose pathway.

The imposition of excessive biotic and abiotic stress to plants can increase the amount of Reactive Oxygen Species (ROS) and cause critical damage to plant cells (Hasanuzzaman et al., 2020). To regulate ROS in cells, plants have developed enzymatic and non-enzymatic antioxidative defense systems in different cell parts (Ishikawa & Shigeoka, 2008). Among these, ascorbate (AsA) is the most abundant water-soluble antioxidant with multiple functions in metabolism, electron transport, control of the cell cycle, and the response of plants to pathogens and biotic stress (Davey et al., 2000; Smirnoff, 2000). Four pathways exist for AsA biosynthesis, with the D-mannose/L-galactose pathway the dominant (Lorence et al., 2004). The L-galactose pathway is regulated by a number of genes which express differentially during the oxidative stress response of a plant against insects and pathogens (Urzica et al., 2012; Lianthanzauva et al., 2020).

We selected the GDP-L-galactose gene (*GGP2*) as our target gene for the L-galactose pathway.

*GGP2* plays an important role in tomato defense responses against abiotic stress and pathogenic infection (Yang et al., 2017); while conversely, deficiency in levels of *GGP2* leads to increased stress susceptibility of tomato plants (Alegre et al., 2020).

##### Mitogen-activated protein kinase pathway.

In all eukaryotic cells, the mitogen-activated protein kinase (MAPK) cascade is one of the major defense pathways involving the transduction of extracellular stimuli into intracellular responses (Zhang & Klessig, 2001). MAPK activation can facilitate signal translocation to the nucleus where, through phosphorylation and activation of transcription factors, gene expression is modulated (Neill et al., 2002). MAPKs are involved in plant signal transduction in response to stress signals from pathogens, drought, cold, wounding, O_3_, ROS, and hormone stimuli (Moon et al., 2003; Mittler et al., 2004). Studies have shown the role of MAPK signaling in plant defense against herbivorous insects (Kandoth et al., 2007; Wu et al., 2007; Wu & Baldwin, 2010), and in fruit response against pathogens and other stresses (Blanco-Ulate et al., 2013; Zhang et al., 2020a; Zhang et al., 2020b). The target genes associated with MAPKs signalling pathway in tomato are *LeMPK1, LeMPK2* and *LeMPK3*.

In tomato, *MPK1*, *MPK2*, and *MPK3* genes have been shown to play an essential role in the wound response signalling pathway and increased plant resistance against *M. sexta* larval herbivory (Kandoth et al., 2007). Conversely, inhibition of tomato *MPK1*, *MPK2*, and *MPK3* genes suppressed tomato fruit defense signaling pathways and increased fruit susceptibility to *B. cinerea* infestation (Zheng et al., 2015). In a broader study on tomato plants, *LeMPK1, LeMPK2* and *LeMPK3* genes were activated in response to stress caused by the wound-signalling peptide systemin, oligosaccharides elicitors, and fungal toxin fusicoccin (Holley et al., 2003; Higgins et al., 2007).

##### Lipoxygenase (LOX).

The LOX genes play important roles during seed germination, plant growth, and in stress response (Porta & Rocha-Sosa, 2002). LOX catalyzes the initial reaction in the biosynthesis and metabolism of jasmonic acid by inserting molecular oxygen into position 13 of a-linolenic acid (a-LeA) (Christensen et al., 2013). Jasmonic acid (JA) itself plays an important role in mediating anti-herbivore defense responses in plants (Howe & Jander, 2008). The LOX family genes have been comprehensively studied in different plant species, including tomato (Mariutto et al., 2011), due to their functions in various physiological and molecular events (Viswanath et al., 2020), including their key role in plant defense response against herbivores and pathogens (Park et al., 2010; Vellosillo et al., 2013).

The target genes associated with LOX defense pathway are *LOXB* and *LOXD*.

In tomato plant, the expression level of the chloroplast-targeted LOX gene, *LOXD,* is rapidly induced by leaf wounding (Heitz, Bergey & Ryan, 1997), whereas the *LOXB* gene is expressed only in seeds or fruits (Ferrie et al., 1994). Antisense suppression of tomato *LOXB* caused JA production deficiency in transgenic tomato fruit (Kausch et al., 2012). Similarly, suppression of *LOXD* severely compromised tomato resistance to *H. armigera* and *B. cinerea* (Dıaz, Ten Have & Van Kan, 2002; Flors et al., 2007; Shen et al., 2014).

#### The GABA signalling pathway

Gamma-Aminobutyric Acid (GABA) is a non-protein, four-carbon amino-acid that occurs naturally in microorganisms, plants, and animals which has various metabolic and physiological functions (Ramos-Ruiz, Martinez & Knauf-Beiter, 2019). These functions include acting as an endogenous signalling molecule in the regulation of plant growth and development (Renault et al., 2011), and being an important component in the regulation of carbon/nitrogen metabolism (Bouche & Fromm, 2004). One of the main roles of GABA accumulation in plants is to increase plant resistance to insect herbivory (Bown & Shelp, 2016). GABA, synthesized from Glutamate decarboxylase (GAD) (Shelp, Mullen & Waller, 2012), is a jasmonic-independent pathway induced rapidly after the wounding of plant tissue and cell disruption by feeding insects (Scholz et al., 2015). GAD activation and GABA accumulation due to disruption of cell structure contribute to constitutive and induced direct-defenses against invertebrates (Bown & Shelp, 2016).

The target gene from the GABA pathway is *LeGAD2*.

Overexpression of Glutamate decarboxylase 2 (*LeGAD2*) gene in transgenic tobacco plants reduced feeding by tobacco budworm larvae (MacGregor et al., 2003). The same study supported the hypothesis that mechanically-induced GABA accumulation contributes a resistance mechanism against invertebrate pests, but this may be dependent on changes in the level of gene expression of proteinase inhibitors or other defense products (Kessler & Baldwin, 2002). In tomato plant, silencing of the *GAD2* gene increased the susceptibility of the plant to bacterial (*Ralstonia solanacearum*) infestation (Wang et al., 2019).

### The phenylpropanoid pathway

Phenylpropanoids are a large class of plant secondary metabolites that are widely distributed in the plant kingdom (Deng & Lu, 2017). Phenylpropanoids mainly include phenolic acids, stilbenes, coumarins, monolignols, and flavonoids (Vogt, 2010; Liu, Osbourn & Ma, 2015). These metabolites have crucial roles in plant development by acting as essential components of cell walls, protectants against UV radiation, phytoalexins against herbivores and pathogens, and floral pigments to mediate plant–pollinator interactions (Seal et al., 2004; López-Ráez et al., 2010; Mandal, Chakraborty & Dey, 2010; De Oliveira et al., 2015). Many phenylpropanoid compounds are induced after wounding or herbivore feeding (Bernards & Båstrup-Spohr, 2008) and participate in the establishment of plant resistance (Vogt, 2010).

The target gene from the phenylpropanoids pathway in tomato tissue is *CCoAOMT*.

The caffeoyl-CoAO methyltransferase gene, *CCoAOMT,* was recorded to be involved in production of coumarin and lignin in plant tissue during plant-pathogen interactions (Do et al., 2007; Kai et al., 2008). In tomato fruit, *CCoAOMT* was found to contribute in the biosynthesis of aromatic compounds and lignin in response to pathogen attack and wounding (Miao et al., 2008).

### Plant induced defense genes (Anti-nutritional activity)

Plant Polyphenol Oxidase genes (PPOs) are distributed widely in different plant tissues and their discoloration effects in damaged and diseased plant tissue have been known for many years (Tran, Taylor & Constabel, 2012). There is strong evidence of constitutive and induced expression patterns of these genes associated with plant defense against pathogens and insects (Thipyapong et al., 2004; Bhonwong et al., 2009). In tomato plant, the PPO gene family consists of seven members: PPO A, Á, B, C, D, E and F (Newman et al., 1993). These PPO gene members are differentially expressed in vegetative and reproductive tissues of tomato in response to biotic and abiotic stressors (Thipyapong, Joel & Steffens, 1997; Thipyapong, Hunt & Steffens, 2004).

The target genes from the PPO family in tomato tissue are *SlPPO1-2*.

The *SlPPO1-2* genes (are also known as *ppo1-2*, *PPO1-2* and *slPPO1-2)* are the *S. lycopersicum* polyphenol oxidase genes (Kampatsikas, Bijelic & Rompel, 2019). *PPO* gene activity is associated with tomato resistance against phloem-feeding and leaf-chewing insects and also pathogens (Ryan, Gregory & Tingey, 1982; Stout et al., 1998). *PPO1* gene overexpression increased tomato plant resistance against *S. litura* larvae (Thipyapong et al., 2004; Mahanil et al., 2008). Both *PPO1* and *PPO2* genes were highly expressed in tomato leaves infested by *Alternaria solani* fungi (Salim et al., 2011).

### Proteinase inhibitor

Plant Protease Inhibitors (PIs) are small proteins that are predominantly present in plant storage tissues, but they have been also found in aerial plant parts (Rehman et al., 2017). Plant PIs are classified as serine proteinase inhibitors, alpha-amylase/trypsin inhibitors, potato type I and type II proteinase inhibitors, serpins, and squash inhibitors (Birk, 2003; Damle et al., 2005). In plant vegetative tissue PIs are induced by insect wounding of plant tissue and play a substantial role in inhibiting folivory (Telang et al., 2009; Chen et al., 2014). Protease inhibitors I and II are also well-known markers of JA mediated defense response in tomato plants and have an anti-nutritive role to feeding herbivores by decreasing the digestibility of dietary protein (Farmer & Ryan, 1992; Felton, 2005). The proteinaceous alpha-amylase inhibitors are accumulated in plant tissues in which they can act as defensive proteins against an insect-herbivore’s digestive alpha-amylases (Franco et al., 2002).

The selected genes, which are responsible for the activity of proteinase inhibitor and alpha-amylase inhibitor, are *PII and CEVI57 (PI-II),* and *a-AIs1*.

*Solanum lycopersicum* wound-induced serine-type proteinase inhibitor I and II (PII and CEVI57, or PI-II), exist in many *Solanaceae* (Bryant et al., 1976; Pearce, Johnson & Ryan, 1993; Fan et al., 2020). Both proteinase inhibitor I and II genes are upregulated in response to mechanical wounding and pathogen attack in tomato plant (Xu et al., 2001; Hamza et al., 2018; Zhang et al., 2020a; Zhang et al., 2020b). Expression of potato inhibitor-II (Pin-II) gene in tobacco plants decreased *M. sexta* larval growth (Johnson et al., 1989); while silencing the *PI* gene in transgenic potato plants increased *Leptinotarsa decemlineata* and *Spodoptera exigua* larval weight (Ortego et al., 2001). The expression of the *PIN2* (proteinase inhibitor II) in both mutant and wild tomato plants was influenced by *Helicoverpa zea* feeding (Tian et al., 2014). Alpha-amylase inhibitor 1 (*a-AIs1*) in tomato negatively impacts a feeding herbivore’s digestive enzymes (Da Lage, 2018). The alpha-amylase inhibitor level was significantly upregulated in damaged leaves of *Amaranthus* by *M. sexta* larvae in comparison to control leaves (Sánchez-Hernández et al., 2004).

### Plant resistance *R* genes

Resistance (R) genes are responsible for the plant’s innate immune system (Dangl & Jones, 2001). Most R genes encode proteins characterized by the existence of a central nucleotide-binding site (NBS), leucine-rich repeats (LRRs), and a variable amino-terminal domain (Takken, Albrecht & Tameling, 2006). The amino-terminal domain determines signalling specificity, while the LRRs are mainly involved in recognition (Martin, Bogdanove & Sessa, 2003). These proteins are distributed across most plant taxa, with the main function being to detect infection by specific pathogens and pests in plant tissue (Chisholm et al., 2006).

The selected gene from this group introduced was *Mi−1.1*.

The NBS-LRR gene, *Mi-1*, is involved in tomato plant resistance against three root-knot nematodes species, potato aphids, tomato powdery mildew and whiteflies (Vos et al., 1998; Nombela, Williamson & Muñiz, 2003; Seifi et al., 2011).

### Primer design

The PCR primers for genes were designed using the Primer-BLAST (NCBI) online tool which combines BLAST with global alignment algorithm to ensure full primer-target alignment while being sensitive enough to detect targets with a noticeable number of mismatches to primers (Ye et al., 2012). The following criteria were considered when designing primer pairs: (i) the annealing T_m_ (melting temperature) should be minimum 58 °C and maximum 62 °C; (ii) the PCR product size must be between 120 to 250 bp; (iii) maximum Poly-x should be 3. For both tomato and *B. tryoni* larvae, mRNA FASTA sequences were used as a PCR template. Each designed primer was tested by inputting to the Primer-BLAST and checking the output gene.

## Results

### Primer check

#### RNA extraction and cDNA library synthesis

Snap frozen tissue of tomato and *B. tryoni* larvae were homogenized by Qiagen TissueLyser II (Retsch) in TRIzol reagent. RNA was extracted from infested tomato and larvae separately using the Isolate II RNA Mini Bioline Kit with a subsequent DNAse treatment using the Turbo DNA-free kit. The quality and quantity of total RNA were checked by running samples on 1.5% denaturing agarose gel and to ensure DNA was absent a Nanodrop was used. The SensiFAST cDNA synthesis kit (BIO-65053) was used to synthesize a cDNA library by adding 15 µl of extracted RNA, 4 µl of TransAmp buffer and 1 µl of reverse transcriptase enzyme. The master mix was placed in the thermal cycler and the cycling conditions were those provided with the kit.

#### Primer check by qPCR analysis

The PCR primer pairs designed for tomato and *B. tryoni* were also tested in qPCR reactions with cDNA of tomato fruit and *B. tryoni* larvae as the experimental samples, respectively. As negative controls, we used No Template Control (NTC) and no-primer control reactions with two technical replicates. qPCR was performed using the SensiFast SYBR No-ROX Kit (BIO-98020). For testing each PCR primer pair, 10 µl of SensiFast SYBR, 0.8 µl of each forward and reverse primers, 0.5 µl cDNA and 7.9 µl H_2_O were used with the final volume of 20 µl. We used LightCycler^®^96 Instrument (Roche) by adjusting two steps cycling and melting: 1 cycle (polymerase activation) in 95 °C for 2 min and 40 cycles in 95 °C in 5 s for denaturation and 60–65 °C in 15–30 s for annealing/extension. The cycle quantification of each target gene were checked (Table 1) and genes with a high cycle threshold (>34) were removed from further analysis. Genes with high NTC cycle threshold (>33) were acceptable for inclusion in the further study. The final PCR primer pairs that were selected in this study are shown in Table 2.

**Table 1:** Mean qPCR primer check results of primer pairs with and without *Bactrocera tryoni* larval tissue cDNA or tomato fruit tissue cDNA. Two technical replications were carried out and the cycle quantification analysis was done in LightCycler^®^ 96, version 1.1.0.1320, Roche. Genes with NTC Cq mean above 33 were acceptable for inclusion in the further study.

Gene name	Experimental Sample		No Template Control (NTC)	
	Cq mean	Cq error (SD)	Cq mean	Cq error
***B. tryoni***				
*GSTD1*	21.04	0.01	–	–
*GSTT1*	18.51	0.00	–	–
*GSTT7*	27.01	0.16	–	–
*ESTF*	24.79	0.08	–	–
*EST1*	29.84	0.18	–	–
*SUR*	28.12	0.19	–	–
*ABCG1*	25.76	0.07	–	–
*ABCA3*	23.00	0.01	–	–
*L259*	25.36	0.02	37.54	0.00
*MDR49*	24.97	0.04	–	–
*CP6A9*	27.98	0.01	39.28	0.00
*CP313*	24.39	0.01	–	–
*CP134*	24.19	0.05	–	–
*CP4D8*	27.80	0.23	37.91	0.00
*CP6G1*	25.07	0.03	–	–
*C12E1*	21.44	0.02	–	–
*CP6T1A*	28.24	0.01	37.16	0.00
*C12C1*	24.28	0.01	37.00	0.23
*CP6T1B*	28.16	0.11	32.96	1.29
*C12B2*	22.29	0.03	37.78	0.98
*C12B1*	26.79	0.01	–	–
*CP306*	27.16	0.02	–	–
*CP304A*	27.74	0.11	–	–
*C6A14*	23.34	0.05	37.83	0.91
*C4AC2*	27.24	0.01	30.82	0.08
*CP4S3*	23.99	0.05	38.13	1.02
*CP132*	24.01	0.02	–	–
*CP316*	28.08	0.03	36.24	0.08
*CP304B*	26.93	0.02	–	–
*CP6G2*	26.66	0.08	38.62	0.00
**Tomato fruit**				
*PORK1*	25.22	0.08	37.34	0.00
*SIPO1*	33.57	1.10	–	–
*SIPO2*	33.10	0.01	–	–
*LeRK1*	21.90	0.02	–	–
*PIIF*	33.43	0.04	–	–
*CEVI57*	18.88	0.22	–	–
*LeMPk1*	21.97	0.09	–	–
*LeMPK2*	20.21	0.18	–	–
*LeMPK3*	18.44	0.01	38.22	0.00
*GGP2*	19.37	0.20	–	–
*Mi_1.1*	26.15	0.00	35.83	0.92
*LOXB*	16.23	0.06	36.95	0.00
*LOXD*	17.41	0.00	35.32	0.74
*CCoAOMT*	18.77	0.11	–	–
*LeGAD2*	20.08	0.06	–	–
*a-AIs1*	30.23	0.30	37.36	0.00

**Table 2:** A list of genes and their PCR primers developed for studying the fruit induced-defense/frugivorous insect-detoxification interactions occurring between *Bactrocera tryoni* larvae feeding in tomato fruit.

***Bactrocera tryoni*** **detoxification pathways genes and primers**		
**Gene** **symbol**	**Forward sequence** **5′–3′**	**Reverse sequence** **5′–3′**
*GSTD1*	GCCGATTTCACCACGTATGC	GCGTGTATCGCTGAAACGTC
*GSTT1*	TTAGCACCATAGACGTGGCG	TGG GCAATACTGCGGAACTT
*GSTT7*	TGGCCGGTGATCAGTTGAAA	GCTGATCGACCATAGCACGA
*ESTF*	AGCTAAACCTTCCACCACGG	CACCCATTGCAAAGCCAGAC
*EST1*	CGCTGTTTACGCATTCCTCG	AGCGGACGCATACTCATAGC
*SUR*	TTGCTCAAGGCAAAGCGAAC	CATCGTCATCCGTCTGCTCA
*ABCG1*	TTCTTTGTCGGTGCTACGCT	ATGGGCGTTCCAAGCCATAA
*ABCA3*	GGGAATAGCGATTGCGGGTA	CGCTTCTTCCATGTGATGCG
*L259*	CAGGAGCCAGCACGTAAAGA	GGTCCAATGACGGCCACTAA
*MDR49*	TGAGGCAACCTCGGCTTTAG	CCGAGCGCATAAGTTCAACG
*CP6A9*	GTATCGCTTGCAACTCGCTG	CGCACGATGCGCATAAAGAA
*CP313*	AACACTTCAAACCGGAGGCA	CTCCAGCTGACACAACGGAT
*CP134*	AGGGCATTTCGATTGGCAGA	TCACCCGCATCGTTTCGTTA
*CP4D8*	ATTTACTCGCACGCCATCCA	CGGCACACTGGGATAGAGAC
*CP6G1*	TGGACGAAGTGTTGCGCTTA	GGATCGAAAGTGTCCGGGTT
*C12E1*	ATGTGGACTTGGAGAACGCA	TCCATTTCCCGAATGGCAGT
*CP6T1A*	TGCATAATCATGCGCTGCTG	GTCTCCAGCTTACCGCCAAT
*CP6T1B*	CGCGCACATCTTTACTCAGC	GCCAGTAACAAGAAAGCGGC
*C12B2*	CAGCTTTCGGATGTTGCGAG	ACCGGCCAGATGGTTTCATT
*C12B1*	TACGCACACTGCCGAAAGAT	TTCCGGACAAGCACTCTCAC
*CP306*	CCTGCTCGCGCTATTAGTCA	TTCAAGAATTCCCGCACCGA
*CP304*	AGCGTCGTGCTGACGATTAT	GTATGCCCATTCGCGTGTTC
*C6A14*	ACACTGCGGAAATACACGGT	CGAAACGATCGGGTTCAGGA
*CP4S3*	AAGCGCTGAAGGTACTGCAT	AAGTGTCGACTTCTTCGCGT
*CP132*	AGCACACCTCTTCAATCCCG	CTGCGATCTCAGCATAACGC
*CP316*	AATCGGTTCGGTGCAGAAGT	ATGATCTGCGCTGTGTAGCA
*CP304*	TGAGGTCGTAGGTAGAGGGC	GCTCCGTGTCTACCAATGCT
*CP6G2*	CGCGCTGTGTTCAAGTTCAG	CGCAGAAACTCGGTAGAGGT
**Tomato defensive pathways genes and primers**		
*PORK1*	AGACCCTCAATGAAAGAGGTA	GGTGGAGCTAGAAGTGAGACA
*slPPO1*	GTGGACAGGATGTGGAACGA	CTTCTTGGTGTCCAGGCAGT
*slPPO2*	AGTTGTTGCCCTCCTGTACC	CCCTCATTCGACTCGTAGCC
*LecRK1*	CTTTGCAGGCATCGTGCTTT	GCGCAAAGGTGAAGGGATTG
*PIIF*	TGGTGTACCAACAAAGCTTGC	GCATTTGTACAACAAAGCCCA
*LeMPK1*	GATGGTTCCGTTCCGCAAAC	GAACCTGCCACCATGGCTTA
*LeMPK2*	GCGCTTGCTCATCCTTACCT	AATCCAACAGCAAACGAGCG
*LeMPK3*	CGCCCTTACGAAGGGAGTTT	ACTTTAGCCCACGGAGAAGC
*GGP2*	CCTCCACTTCCAGGCGTATT	GCATCAGACAAATCACGGGC
*Mi-1.1*	AAAGCTCACCAGTGGATCGG	CCATGCACGAAGGTCGAAAC
*LOXB*	GCGTTTAAGGCTTTGTGCGA	GTAGGCCTTGACCATCCGTT
*LOXD*	GCAGATCGCTAAAGCACACG	GCGCTTAACTGCCTATGTGC
*CCoAOMT*	ACCAAATGATTGACGACGGC	TCCGTTCCAAAGGGTGTTGT
*LeGAD2*	TGAGCCCTGAGAAAGCTGTG	GGAGTGTCCCACCCTGTTTC
*a-AIs1*	AAGTGCCTCACCAACACCAT	CAGAATTCGTCGCGGATGGA

**Table 3:** Mean qPCR primer check results of primer pairs for selected putative detoxification genes in *Bactrocera tryoni* and induced-defence mechanism genes in tomato fruit. For *B. tryoni larvae*, *RS10B, RK18A, RT15, RT14* genes and for tomato *FPPS1, IDI1* genes used as housekeeping genes (internal control). The qPCR template was cDNA for *B. tryoni* larvae or tomato tissue. The Cq means calculated from two technical replications and the cycle quantification analysis was done in LightCycler^®^ 96, version 1.1.0.1320, Roche.

Gene name	Unpicked status			Picked status		
	Cq mean			Cq mean		
	Rep 1	Rep 2	Rep 3	Rep 1	Rep 2	Rep 3
***B. tryoni***						
*GSTD1*	19.66	20.07	19.19	20.23	20.38	19.96
*GSTT1*	17.10	17.71	17.45	17.93	17.79	18.86
*GST7*	25.64	25.34	25.00	25.67	25.90	25.05
*ESTF*	23.82	24.10	23.22	23.91	23.81	24.21
*EST1*	28.63	28.39	28.25	28.38	28.42	28.26
*SUR*	27.10	26.81	26.05	27.36	27.17	27.34
*ABCG1*	24.46	24.71	24.84	25.01	25.18	25.68
*ABCA3*	22.69	22.82	21.74	22.27	22.78	22.39
*L259*	24.81	25.07	24.70	24.51	24.64	24.53
*MDR49*	24.06	25.17	22.37	24.15	24.16	23.29
*CP6A9*	26.98	27.33	26.84	26.80	27.08	26.53
*CP313*	22.66	23.67	21.76	22.99	23.79	22.27
*CP134*	27.25	27.19	24.71	26.77	26.95	25.07
*CP4D8*	28.46	29.37	29.54	27.91	29.18	26.57
*CP6G1*	27.03	27.09	26.98	27.75	27.71	27.08
*C12E1*	23.12	22.76	21.52	22.60	22.77	21.69
*CP6T1A*	28.71	29.11	28.43	28.34	29.32	27.43
*CP6T1B*	28.72	28.86	28.57	28.01	29.08	27.09
*C12B2*	22.96	22.24	21.03	22.05	22.15	22.03
*C12B1*	26.86	27.17	25.89	26.92	26.88	26.00
*CP306*	25.65	25.84	25.30	25.13	25.76	25.93
*CP304A*	28.68	28.49	29.74	28.05	28.93	27.42
*C6A14*	24.44	23.56	21.97	23.83	23.47	22.85
*CP4S3*	24.70	23.96	23.80	24.11	24.91	25.08
*CP132*	23.03	22.59	21.98	22.71	22.30	22.00
*CP316*	27.65	27.64	26.09	27.34	26.97	26.74
*CP304B*	27.68	28.01	28.03	27.32	28.55	27.09
*CP6G2*	27.43	27.60	26.89	27.06	27.91	26.84
**HK genes**						
*RS10B*	14.60	14.49	14.12	14.42	14.87	14.84
*RK18A*	13.92	13.76	13.48	13.94	14.11	13.91
*RT15*	19.59	19.39	19.41	19.25	19.56	20.28
*RT14*	19.46	19.06	18.77	19.26	19.37	19.50
***Tomato fruit***						
*PORK1*	24.56	25.10	25.4	25.31	26.13	27.01
*SIPO1*	30.63	29.33	30	31.92	30.81	30.04
*SIPO2*	30.55	29.86	28.87	30.18	31.53	30.52
*LeRK1*	20.90	20.94	21.58	21.34	22.14	22.70
*PII*	21.33	20.74	18.89	21.01	21.48	29.22
*LeMPK1*	21.73	21.48	21.47	21.91	22.41	24.42
*LeMPK2*	19.44	19.72	19.72	20.11	20.10	21.10
*LeMPK3*	18.94	18.56	18.74	18.99	19.41	20.29
*GGP2*	19.01	19.48	19.07	19.33	19.38	21.01
*Mi_1.1*	26.53	26.02	27.06	27.74	27.61	29.83
*LOXB*	15.56	17.04	16.88	15.29	14.83	14.41
*LOXD*	17.63	17.55	18.79	18.18	20.06	20.71
*CCoAOMT*	19.10	19.96	19.84	19.64	20.21	21.62
*LeGAD2*	19.37	19.89	19.49	20.06	20.27	23.93
*a-AIs1*	24.60	29.13	25.71	29.20	32.32	30.03
**HK genes**						
*FPPS1*	18.22	19.70	18.46	18.40	18.78	19.63
*IDI1*	18.00	18.60	18.17	18.39	18.04	18.70

#### Primer consistency in experimental samples

To demonstrate the Cq consistency of the PCR primer pairs, the qPCR results from three replicates of our subsequent study have been presented in Table 3. The results were obtained from phenotypic and molecular studies to identify tomato fruit induced defense responses against *B. tryoni* larvae. The experiment was conducted under semi-natural conditions (glasshouse) while tomatoes were still on the plant. Fourty fruit from each of the two different cultivars and two different ripening stages were inoculated with 40 *B. tryoni* neonate larvae. After inoculation, half of the fruit were picked immediately and kept in the same condition as unpicked fruit. Inoculated fruit were then dissected at two different time points (48 hr and 120 hr) to reflect the two different larval stages under normal developmental conditions. Surviving *B. tryoni* larvae and infested tomato tissue from each of the fourty replicates were transferred to 2.00 ml microtubes separately and then snap frozen using liquid nitrogen and kept at −80 °C until required for RNA extraction.

Here in Table 3, the cycle threshold of three replicates from unpicked and picked treatments (tomato tissue and surviving larvae) at 48 hr timepoint shows the primer consistency in candidate genes under the experimental conditions. The amount of tissue in each of the three replicates included the tissue collected in 2.00 ml microtubes for tomato and 20–25 larvae for *B. tryoni.*

## Conclusion

Through a combined worked-example and literature review, this paper has identified genes known to be associated with induced-defense against herbivores and pathogens in tomatoes, and genes putatively associated with detoxification in *B. tryoni* based on their known action in other insect herbivore systems. Applied to *B. tryoni* larvae and tomato fruit harvested under different experimental conditions, the genes selected have been shown to respond based on the predictable patterns from the literature (S Roohigohar, AR Clarke, PJ Prentis, 2021, unpublished data). Of 30 selected genes for *B. tryoni* larvae, two genes (*C4AC2* and *C12C1*) were excluded from our study due to PCR primer failure (Table 1) or high Cq in most of replicates. In tomato, one gene (*CEVI57*) from 16 selected genes was excluded due to no Cq in most of replicates. The PCR primers designed are specific for *B. tryoni* and tomato, but the approach followed is directly transferable to other systems so long as there is already at least some genomic resources, at a minimum an annotated transcriptome.

The gene selection process for *B. tryoni* larvae developed here is novel in insect frugivory research. In contrast, the much more straight-forward gene selection process for tomato fruit (Fig. 2) shows the advantage of having expanded functional genomic studies which are now common in plant pathology. Plant protection entomologists are clearly still lagging with respect to their plant pathology colleagues in this field.

Untargeted molecular approaches, such as comparative transcriptomics, provide important insights into the overall changes in gene expression associated with two or more states, such as larvae growing in different fruit types (Corrado et al., 2012). However, more quantitative candidate gene studies, such as RT-qPCR approaches, are also needed if the intent of the research is to create resistant fruit genotypes. For a fruit fly/fruit system, knowing when and where (i.e., on what cellular or metabolic pathway) larvae are most challenged by plant defenses, and similarly when and how the fruit are challenging the larvae, is fundamental to any manipulation of the system.

##  Supplemental Information

10.7717/peerj.11762/supp-1 Supplemental Information 1 Detoxification genes which were excluded from a molecular biology study focusing on *Bactrocera tryoni* larvae and why they were discarded

## Additional Information and Declarations

### Data Availability

The following information was supplied regarding data availability: The raw data is available at Zenodo: Shirin Roohigohar. (2020). Gene selection for studying frugivore-plant interactions: a review and an example using Queensland fruit fly in tomato. http://doi.org/10.5281/zenodo.4267156.
