# Supplementary material for: Gene selection for studying frugivore-plant interactions: a review and an example using Queensland fruit fly in tomato
Source: PeerJ. 2021 Aug 5;9:e11762. doi: 10.7717/peerj.11762 (PMC8359797; doi:10.7717/peerj.11762)
Supplement: Supplemental Information 1 [file peerj-09-11762-s001.docx]

| Gene name | Family | The reason for exclusion |
| --- | --- | --- |
| *GSTEE* | Glutathione S-transferases | Uncharacterized protein |
| *GST1D* | Glutathione S-transferases | Uncharacterized protein |
| *GSTS1* | Glutathione S-transferases | <80% identity match |
| *EST2* | Carboxylesterase | Uncharacterized protein |
| *ABCG8* | ABC transporters | Uncharacterized protein |
| *CP9B1* | Cytochrome P450 | Uncharacterized protein |
| *C4AD1* | Cytochrome P450 | Uncharacterized protein |
| *C28D1* | Cytochrome P450 | <80% identity match |
| *CP4E2* | Cytochrome P450 | Uncharacterized protein |
| *C12A2*  *C4AC2*  *C12C1* | Cytochrome P450  Cytochrome P450  Cytochrome P450 | Uncharacterized protein  PCR primer failed  Untargeted/absent Cq |
